# 25 Impact of early feeding on the occurrence of juvenile idiopathic arthritis

**DOI:** 10.1093/rheumatology/keac496.021

**Published:** 2022-10-07

**Authors:** Emna Rabhi, Wafa Triki, Hanene Ferjani, Dorra Ben Nessib, Kaouther Maatallah, Dhia Kaffel, Wafa Hamdi

**Affiliations:** 1 Faculty of Medicine of Tunis, Beb Saadoun, Tunisia; 2 Kassab Orthopedics Institute, Rheumatology Department, Ksar Said, Tunisia

## Abstract

**Background:**

Juvenile idiopathic arthritis (JIA) is considered to be an autoimmune disease, which is a result of an immune reaction caused or triggered by environmental factors in a genetically susceptible individual. However, interactions between early feeding and risk Juvenile idiopathic arthritis (JIA) remains largely unexplored.

**Objectives:**

To determine the impact of early alimentation on the occurrence of JIA in Tunisia.

**Methods:**

We conducted a case-control study including 26 children with JIA fulfilling the international league against rheumatism (ILAR) 2010 criteria compared with a homogeneous control group of 21 children hospitalized in a paediatric orthopedic unit for traumatic reasons and healthy for any chronic inflammatory rheumatism. Parents of patients were asked after having their consent to participate. We collected the following data: Type of JIA, juvenile disease activity score (JADAS), type of feeding, total duration of breastfeeding, duration of exclusive breastfeeding and age at introduction of gluten. Patients were divided into two groups: group 1 (G1): JIA patients, group 2 (G2): control group. Data were analyzed using the SPSS statistical package. A *p*-value < 0.05 was considered significant.

**Results:**

Forty-seven children (26 diagnosed with JIA and 21 controls) with a mean age of 10.07 years ± 4.96 were enrolled. JIA subtypes were in decreasing order of frequency: oligoarticular (45.8%), enthesitis-related arthritis (20.8%), undifferentiated JIA (12.5%), seronegative polyarticular JIA (8.3%), juvenile psoriatic arthritis (8.3%), and seropositive polyarticular JIA (4.2%). The mean JADAS was 6.966 ± 5.38. There was no significant difference between the G1 and G2 with regard to the duration of breastfeeding (12.23 ± 3.8 months *vs* 8.14 ± 4.1 months, *p* = 0.09), exclusive breastfeeding (3.8 ± 3.6 *vs* 4.1 ± 2.62, *p* = 0.40) and age at introduction of gluten (G1: 8.48 ± 5.30 months *vs* G2: 7.48 ± 1,2 months, *p* = 0.35)

**Conclusion:**

Our study didn’t show any impact of early alimentation on the occurrence of JIA in Tunisia. However, more studies are necessary to conclude these issues.

